# Lifestyle-related behaviors and health-related quality of life among children and adolescents in China

**DOI:** 10.1186/s12955-020-01657-w

**Published:** 2021-01-06

**Authors:** Zhenzhen Qin, Na Wang, Robert S. Ware, Yugen Sha, Fei Xu

**Affiliations:** 1 Nanjing Municipal Center for Disease Control and Prevention, 2, Zizhulin, Nanjing, 210003 China; 2 Menzies Health Institute Queensland, Griffith University, Brisbane, Australia; 3 Department of Nephrology, Children’s Hospital of Nanjing Medical University, 72, Guangzhou Road, Nanjing, 210008 China; 4 Department of Epidemiology, School of Public Health, Nanjing Medical University, Nanjing, China

**Keywords:** Lifestyle, Behavior pattern, Health-related quality of life, Children, Adolescents

## Abstract

**Purpose:**

To investigate associations of five typical lifestyle-related behavioral risk factors (insufficient physical activity, prolonged screen viewing, deprived sleeping, consumption of fast food and sugar-sweetened beverage) with health-related quality of life (HRQoL) among school students in China.

**Methods:**

Students aged 9–17 years (grades 4–12) were randomly selected from primary and high schools in Nanjing, China, to participate in this cross-sectional study in 2018. The outcome variable, HRQoL, was assessed using the Child Health Utility 9D (CHU9D) instrument and scored from 0 (worst) to 1 (best). Physical activity (including screen viewing and sleeping) and dietary intake were measured using a validated Physical Activity Scale and Food Frequency Questionnaire, respectively. Lifestyle-related behaviors were categorized as sufficient/insufficient or no/yes, and their associations with HRQoL were assessed using mixed-effects linear regression models.

**Results:**

Overall, 4388 participants completed the questionnaire (response rate = 97.6%). Students with insufficient physical activity [mean difference (MD) = − 0.03; 95% confidence interval (CI) = − 0.04, − 0.01], prolonged screen time (MD = − 0.06; 95% CI = − 0.07, − 0.04), insufficient sleeping time (MD = − 0.04; 95% CI = − 0.07, − 0.02), consumption of sugar-sweetened beverage (MD = − 0.02; 95% CI = − 0.03, − 0.01) or fast food intake (MD = − 0.03; 95% CI = − 0.04, − 0.02) reported significantly lower HRQoL scores. When considered additively, each additional lifestyle-related risk factor was associated with an average decrease of 0.03 units (95% CI: − 0.03, − 0.02) CHU9D score.

**Conclusions:**

For Chinese students, HRQoL was positively associated with physical activity and sleep duration, but negatively with screen time and consumption of sugar-sweetened beverage and fast food. Moreover, lifestyle-related behaviors may have an additive effect on HRQoL.

## Background

Health related quality of life (HRQoL) is a broad and multi-dimensional measure of an individual’s perceived physical and mental health, which usually includes self-perceptions regarding disease symptoms or health conditions, side effects, functional status across different life domains, and life quality and satisfaction [1–3]. HRQoL has recently been used to evaluate effects of lifestyle and behavior intervention programs for children and adolescents from a public health perspective [4, 5]. Consequently, it is important to better understand the relationship between lifestyle-related behavior factors measured in population-based lifestyle and behavior interventions and HRQoL in this population.

Lifestyle and behavior patterns, including physical activity (PA), sedentary behavior (SB, indicated as screen time, ST), sleeping time (predicted by total sleep duration, TSD) and fast food (FF) consumption, were identified to be associated with HRQoL among adolescents in Western societies [6–12]. There are positive associations between PA, TSD and HRQoL [6–11], but negative associations of SB and FF consumption with HRQoL [6–10]. Moreover, an Australian study of school students with 5-year follow-up showed that the more lifestyle-related behavior risk factors (low PA, high SB, high FF consumption, high sugar-sweetened beverages (SSB) consumption) participants recorded, the lower HRQoL they reported [12].

China is the most populous country in the world with the largest number of children and adolescents. One Chinese-based investigation, a pilot study conducted by our research team in May of 2013, has examined the associations of PA and TSD with HRQoL among school students, showing a positive association for both risk factors with HRQoL [13]. It is important to further explore the relationship between lifestyle-related behaviors and HRQoL among Chinese students using data from large well-designed population studies. To fill this knowledge gap we conducted a school-based questionnaire survey among students in Nanjing Municipality, China, with the aims: (1) to examine individual associations of PA, ST, TSD, FF consumption, SSB consumption with HRQoL; and (2) to investigate the combined effect of these five lifestyle-related behavior factors on HRQoL.

## Methods

### Study participants and sampling approach

Data were collected as part of a cross-sectional survey, The Built Environment and Chronic Health Conditions: Children (BEACH-Children) Study, which was conducted among primary and high school students during May and June of 2018 in Nanjing, a mega-city in eastern China. The BEACH-Children Study had three broad objectives: to explore the association between built environment attributes and PA/obesity; to examine the relationship between lifestyle-related behavioral patterns and HRQoL; and to investigate health literacy prevalence and its associated characteristics.

The study city, Nanjing, had more than eight million registered inhabitants residing in 12 administrative districts in 2018 [14].There were approximately 667,300 enrolled primary, junior and senior high school students in the 2017–2018 academic year, with approximately 40 students per class [15]. BEACH-Children study participants were those aged 9–17 years from school grades 4–12 (primary, grade 4–6; junior, grade 7–9; senior, grade 10–12) within all 12 districts.

The sample size estimation and participants’ selection have been described in detail elsewhere [16]. Briefly, the sample size for the BEACH-Children Study was determined so that the study would have sufficient statistical power to simultaneously test the three original hypotheses, accounting for the multi-stage cluster sampling approach and the cross-sectional study design. Then, it was estimated that approximately 3900 participants should be recruited. Potential participants were identified using random digits. First, one primary, junior and senior high school was randomly selected from each of the 12 districts. Next, one class from each target grade in the chosen schools was selected, giving 108 selected classes from 36 schools in 12 districts. All students within each selected class were invited to participate.

We obtained written informed consent from both participating schools and students’ parents/ guardians. This study was approved by The Academic and Ethics Committee of Nanjing Municipal Center for Disease Control and Prevention, China (2017006). All identifiable participant information was removed before data analysis.

### Data collection

Participants completed a questionnaire which collected information on demographic and social characteristics. Specific sub-scales were used to gather data on HRQoL, PA, ST, TSD, and FF and SSB consumption, academic performance and general health status. On the designated questionnaire day, participants were asked to complete the survey within their regular classroom. To warrant the maximal response rate, (1) we discussed the specific survey date with participating schools one by one in order to have students engaged in as possible; and (2) one classroom teacher and two research team members were available within each class to provide on-site assistance upon request. Parental educational attainment was recorded by students and checked by their classroom teachers. In China, when children registered with a school, they were required to provide the school their family’s primary data including home address, parents’ age and educational level, etc. Participants’ body weight and height were objectively measured inside a quiet room by research team members, each reading was taken twice and the mean value was used for analysis.

### Study variables

#### Outcome variable

HRQoL was assessed with the validated Chinese version of Child Health Utility 9D (CHU9D-CHN) [13], whose scoring algorithm was specifically developed for children and adolescents in China [17]. CHU9D is a newly developed preference-based generic instrument of HRQoL, specifically for assessing cost-effectiveness of clinical treatment or/and public health intervention programs particularly for children and adolescents [18]. The CHU9D utility score, ranging from 0 (worst) to 1 (best), was analyzed as continuous variable in our study.

### Explanatory variables

#### Physical activity

The validated Item-specific Physical Activity Scale for Chinese Children and Adolescents (I-PASCA) was applied to gather information on participants’ physical activity in the previous 7 days [19]. The total time for each PA item was calculated by multiplying duration per session and frequency per week. A specific metabolic equivalent (MET) value was designated for each PA item, before each activity was classified as light-intensity (MET: < 3), moderate-intensity (MET: 3–6) or vigorous-intensity (MET: ≥ 6) [20]. The total weekly moderate-to-vigorous PA (MVPA) time (moderate-PA time plus doubled vigorous-PA time) was calculated and converted to daily MVPA time. According to PA recommendation for Chinese children and adolescents, sufficient PA was defined as “at least 60 min/day MVPA plus ≥ 3 days/week muscle/bone-strengthening” [21]. Participants were categorized as “not achieving sufficient PA” or “achieving sufficient PA” for analysis.

### Screen time and total sleep duration

Screen viewing was used to indicate sedentary behavior and was reported as part of I-PASCA. Average daily screen time was calculated and classified as: “without prolonged screen time (viewing screen < 2 h/day)” or “with prolonged screen time (viewing screen ≥ 2 h/day)” [21]. Sleep time was recorded as typically daily total sleep duration using I-PASCA too. Participants were asked to self-report the average sleep hours in total per day (including nap time) they slept in the last 7 days. TSD was categorized as “insufficient” or “sufficient” based on the age-specific guidelines issued by National State Council of China [22].

### Fast food and sugar-sweetened beverages consumption

A validated food frequency questionnaire (FFQ) designed to assess dietary intake specifically for Chinese children and adolescents was used to collect information on food consumption, including FF and SSB, over the previous 7 days [23]. Considering that the mean value of FF and SSB consumption frequency was very small (0.61 serves/week for FF, 1.43 serves/week for SSB), we categorized participants into two sub-groups for analysis: weekly consumed FF/SSB (“Yes”) or did not consume FF/SSB (“No”).

### Simultaneous engagement in risk behaviors

A risk factor index (RFI) was created to examine the joint association of lifestyle-related behaviors with HRQoL. The RFI was computed as the total number of risk lifestyle-related behaviors a participant engaged in. Five typical lifestyle-related risk behaviors/factors were considered: insufficient PA, prolonged ST, insufficient TSD, weekly consumption of FF, and weekly intake of SSB. The RFI ranged from 0 (no risk behaviors) to 5 (all risk behaviors identified).

### Demographic and social characteristics

The following demographic and social characteristics were collected and included in multivariable regression models as covariables: participants’ age, gender, residence, parental educational level and school type. Age was included as a continuous variable, while all other characteristics were treated as categorical variables (gender, boys/girls; residence, rural/suburban/urban; parental educational attainment, ≤ 9 years/10–12 years/13+ years; and school type, primary/junior high/senior high) in the analysis. Body weight status was defined as “underweight/normal”, “overweight” or “obesity” based on body mass index (BMI) recommendations for Chinese school-aged children and adolescents [24].

### Statistical analysis

Summary statistics are presented as mean (standard deviation, SD) or frequency (percentage). Associations between participant characteristics and school type were compared using the chi-square test. Associations between participant characteristics and HRQoL utility scores were compared using Students t-test. To investigate the associations between lifestyle-related behaviors and HRQoL, mixed-effects linear regression models were used and effect estimates were reported as the mean difference (MD) and 95% confidence interval (CI). First univariable models were run, with each lifestyle-related behavior included as the main effect, then multivariate models were run with age, gender, BMI, school type, residence, and parental educational attainment included as covariables. In all models ‘school class’ was included as a random effect to account for possible non-independence of response from children within the same class. A *P* value < 0.05 (two sided test) was considered as significant. The data was double-entered with EpiData 3.1 (The EpiData Association 2008, Odense, Denmark) and analyzed by SPSS version 20.0 for Windows (SPSS Inc., Chicago, IL, USA).

## Results

### Participant characteristics

Of the 4498 students eligible to take part in the study, 4388 completed the survey (response rate = 97.6%). There were no significant differences between those who did and did not complete the questionnaire in terms of gender, grade and residence. Table 1 displays participants’ characteristics and lifestyle-related behavior patterns by school type. Overall, 36.5%, 33.2% and 30.3% of respondents were primary, junior high and senior high school students, respectively; the mean age (SD) was 13.9 (2.5) years; 50.2% were boys.

**Table 1:** Selected characteristics of participants by school type in this study

Variables	Overall (N = 4388)	School type			*P* value^a^
		Primary school (N = 1602)	Junior high school (N = 1455)	Senior high school (N = 1331)	
Age					
Mean (SD^b^)	13.9 (2.5)	11.2 (0.9)	14.1 (0.9)	16.9 (0.9)	
Gender (%)					
Boys	50.2	53.9	50.9	45.1	< 0.001
Girls	49.8	46.1	49.1	54.9	
Residence (%)					
Rural	46.3	44.5	46.5	48.1	0.19
Suburban	22.7	24.5	22.2	21.1	
Urban	31.0	31.0	31.3	30.8	
Body weight status (%)^c^					
Non-overweight	72.3	67.8	73.4	76.6	< 0.001
Overweight	16.7	17.8	16.9	15.2	
Obesity	11.0	14.4	9.7	8.2	
Physical activity (%)^d^					
Sufficient	21.2	29.3	20.5	12.1	< 0.001
Insufficient	78.8	70.7	79.5	87.9	
Screen time (%)					
< 2 h/day	96.3	96.8	95.3	96.7	0.06
≥ 2 h/day	3.7	3.2	4.7	3.3	
Sleeping time (%)^e^					
Sufficient	20.0	23.0	19.0	17.5	< 0.001
Insufficient	80.0	77.0	81.0	82.5	
Sugar-sweetened beverage consumption^f^					
No	67.5	71.0	61.9	69.6	< 0.001
Yes	32.5	29.0	38.1	30.4	
Fast food consumption^f^					
No	67.6	62.9	70.9	69.8	< 0.001
Yes	32.4	37.1	29.1	30.2	

^a^Chi-square test

^b^*SD* standard deviation

^c^Body weight status was categorized based on age- and gender-specific recommendations for Chinese adolescents

^d^Sufficient physical activity refers to at least 60 min/day moderate-vigorous intensity physical activity plus ≥ 3 days/week muscle/bone-strengthening; while insufficient physical activity means less than 60 min/day moderate-vigorous intensity physical activity or having no ≥ 3 days/week muscle/bone-strengthening

^e^Sleeping time: sufficient sleeping time was defined as 10 h/day for children aged 7–13, 9 h/day for children aged 13–16, and 8 h/day for those aged 16–19, based on guidelines for promotion of children and adolescents’ physical activity and fitness by The State Council of China

^f^FF and SSB consumption was classified as “no” or “yes” based on the separate consumption frequency

### Individual associations between risk factors and HRQoL utility scores

The overall mean HRQoL utility score (SD) was 0.78 (0.17). Table 2 shows the association between each of the five lifestyle-related behaviors and HRQoL utility scores. The HRQoL utility score decreased as children aged (from 0.84 ± 0.16 for primary students, to 0.78 ± 0.16 for junior high students and to 0.73 ± 0.18 for senior high students), but increased as parental educational attainment increased (from 0.76 ± 0.17 for junior high or less schooling to 0.78 ± 0.16 for senior high schooling and 0.80 ± 0.18 for college or greater schooling).

**Table 2:** Association of risk factors with HRQoL score based on mixed-effects linear regression analysis

Lifestyle-related behavior	Mean (SD)	Univariable model^d^			Multivariable model^e^		
		Mean difference	95% CI	*P*	Mean difference	95% CI	*P*
Physical activity^a^							
Sufficient	0.82 (0.15)	Ref			Ref		
Insufficient	0.77 (0.18)	− 0.04	− 0.07, − 0.01	< 0.001	− 0.03	− 0.04, − 0.01	< 0.001
Screen time							
< 2 h/day	0.78 (0.17)	Ref			Ref		
≥ 2 h/day	0.73 (0.19)	− 0.05	− 0.09, − 0.01	0.010	− 0.04	− 0.070, − 0.02	0.002
Sleeping time^b^							
Sufficient	0.84 (0.16)	Ref			Ref		
Insufficient	0.77 (0.17)	− 0.10	− 0.15, − 0.05	< 0.001	− 0.06	− 0.07, − 0.04	< 0.001
Sugar-sweetened beverage consumption^c^							
No	0.79 (0.17)	Ref			Ref		
Yes	0.76 (0.17)	− 0.02	− 0.05, 0.01	0.119	− 0.02	− 0.03, − 0.01	< 0.001
Fast food consumption^c^							
No	0.79 (0.17)	Ref			ref		
Yes	0.76 (0.18)	− 0.03	− 0.06, 0.001	0.060	− 0.03	− 0.04, − 0.02	< 0.001

^a^Sufficient physical activity refers to at least 60 min/day moderate-vigorous intensity physical activity plus ≥ 3 days muscle/bone-strengthening; while insufficient physical activity means less than 60 min/day moderate-vigorous intensity physical activity or having no ≥ 3 days muscle/bone-strengthening

^b^Sleeping time: sufficient sleeping time was defined as 10 h/day for children aged 7–13, 9 h/day for children aged 13–16, and 8 h/day for those aged 16–19, based on guidelines for promotion of children and adolescents’ physical activity and fitness by The State Council of China

^c^FF and SSB consumption was classified as “no” or “yes” based on the weekly consumption frequency, separately

^d^Model 1: univariate mixed-effects model with school class as the random effect

^e^Model 2: multivariate mixed-effects linear regression analysis with adjustment for age, gender, school type, residence, parental educational attainment, body weight status and class-level clustering effects

Participants with insufficient PA and TSD reported lower HRQoL utility scores after adjustment for potential confounding variables (MD = − 0.03; 95% CI = − 0.04, − 0.01 and MD = − 0.06; 95% CI = − 0.07, − 0.04 respectively), while participants with excess ST, consumption of FF and SSB recorded lower HRQoL utility scores (MD = − 0.04; 95% CI = − 0.07, − 0.02; MD = − 0.02; 95% CI = − 0.03, − 0.01, and MD = − 0.03; 95% CI = − 0.04, − 0.02 respectively). Similar findings were found among almost all sub-groups stratified by gender, school type and residence ("Appendix 1").

### Combined association between risk factors and HRQoL utility scores

The joint association of the five lifestyle behavior risk factors with HRQoL score was also examined (Table 3). The RFI is a combination of the five separate factors. After adjustment for potential confounders and class-level clustering effects, a negative linear association was identified between RFI and HRQoL score (β = − 0.03; 95% CI: − 0.03, − 0.02). As the number of risk factors increased, HRQoL utility scores steadily decreased, with an average reduction of 0.03 units for each additional risk factor. Similar findings were observed for participants stratified by gender, school type or residence area.

**Table 3:** Adjusted linear associations between risk behavior factors and HRQoL utility scores in this study

Risk factors index^a^	Overall (N = 4388)			Gender						School type									Residence								
	Estimate	95% CI	*P*	Boys (N = 2204)			Girls (N = 2184)			Primary (N = 1602)			Junior high (N = 1455)			Senior high (N = 1331)			Rural (N = 2030)			Sub-urban (N = 997)			Urban (N = 1361)		
				Estimate	95% CI	*P*	Estimate	95% CI	*P*	Estimate	95% CI	*P*	Estimate	95% CI	*P*	Estimate	95% CI	*P*	Estimate	95% CI	*P*	Estimate	95% CI	*P*	Estimate	95% CI	*P*
Continuous variable	− 0.03	− 0.03, − 0.02	*0.000*	− 0.02	− 0.03, − 0.02	< 0.001	− 0.03	− 0.04, − 0.03	< 0.001	− 0.02	− 0.03, − 0.02	< 0.001	− 0.03	− 0.04, − 0.02	< 0.001	− 0.02	− 0.03, − 0.01	< 0.001	− 0.03	− 0.03, − 0.02	< 0.001	− 0.03	− 0.04, − 0.02	< 0.001	− 0.03	− 0.04, − 0.01	*0.002*
1	− 0.03	− 0.07, − 0.002	0.038	− 0.04	− 0.08, 0.01	0.098	− 0.04	− 0.08, 0.01	0.165	− 0.03	− 0.07, 0.02	0.216	− 0.05	− 0.11, 0.01	0.134	− 0.03	− 0.11, 0.05	0.414	− 0.03	− 0.07, 0.01	0.194	− 0.05	− 0.12, 0.02	0.167	− 0.04	− 0.11, 0.04	0.312
2	− 0.07	− 0.10, − 0.04	0.000	− 0.07	− 0.11, − 0.03	0.001	− 0.08	− 0.12, − 0.03	0.002	− 0.05	− 0.09, − 0.01	0.014	− 0.09	− 0.15, − 0.03	0.004	− 0.08	− 0.16, − 0.01	0.036	− 0.06	− 0.10, − 0.02	0.003	− 0.09	− 0.16, − 0.03	0.008	− 0.08	− 0.15, − 0.01	0.025
3	− 0.10	− 0.13, − 0.07	0.000	− 0.09	− 0.13, − 0.05	< 0.001	− 0.11	− 0.16, − 0.06	< 0.001	− 0.08	− 0.12, − 0.04	< 0.001	− 0.11	− 0.17, − 0.05	0.001	− 0.11	− 0.195, − 0.03	0.007	− 0.09	− 0.13, − 0.05	< 0.001	− 0.13	− 0.18, − 0.04	0.002	− 0.10	− 0.17, − 0.03	0.005
Categorical variable																											
4	− 0.11	− 0.15, − 0.08	0.000	− 0.11	− 0.15, − 0.06	< 0.001	− 0.13	− 0.18, − 0.08	< 0.001	− 0.09	− 0.14, − 0.05	< 0.001	− 0.15	− 0.22, − 0.09	< 0.001	− 0.10	− 0.18, − 0.02	0.017	− 0.09	− 0.14, − 0.05	< 0.001	− 0.15	− 0.23, − 0.08	< 0.001	− 0.12	− 0.19, − 0.05	0.002
5	− 0.14	− 0.20, − 0.08	0.000	− 0.13	− 0.20, − 0.05	0.002	− 0.17	− 0.26, − 0.07	< 0.001	− 0.14	− 0.24, − 0.05	0.003	− 0.18	− 0.28, − 0.09	< 0.001	− 0.08	− 0.22, 0.07	0.296	− 0.15	− 0.25, − 0.05	0.005	− 0.22	− 0.39, − 0.04	0.015	− 0.13	− 0.22, − 0.03	0.010

*p* < 0.05 was set as statistical significance (two-sides)

Mixed-effects linear regression analysis with adjustment for for age, gender, school type, residence, parental educational attainment, body weight status and class-level clustering effects

Sufficient physical activity refers to at least 60 min/day moderate-vigorous intensity physical activity plus ≥ 3 days muscle/bone-strengthening; while insufficient physical activity means less than 60 min/day moderate-vigorous intensity physical activity or having no ≥ 3 days muscle/bone-strengthening

Sleeping time: sufficient sleeping time was defined as 10 h/day for children aged 7–13, 9 h/day for children aged 13–16, and 8 h/day for those aged 16–19, based on guidelines for promotion of children and adolescents’ physical activity and fitness by The State Council of China

Sugar-sweetened beverage and fast food consumption was separately categorized into: “no” or “yes” based on the weekly consumption frequency

^a^Risk behavior factors constructing risk factor index refers to insufficient PA, prolonged ST, insufficient TSD, low intake of FF, and high intake of SSB. reference: no risk factor involved

## Discussion

In this school-based study, we investigated the associations between lifestyle-related behaviors and health-related quality of life among school students in regional China. We found that PA and TSD were positively associated with HRQoL utility score, while SB, consumption of FF and SSB were negatively associated with HRQoL utility score. The greater the number of lifestyle behavior risk factors each participant engaged in, the lower the HRQoL score they reported, suggesting an additive effect existed for the five identified behavior risk factors on self-reported HRQoL utility score among school students in China.

In previous studies conducted in Western societies, positive associations between PA, TSD and HRQoL, and negative associations between SB, FF and HRQoL have been documented among children and adolescents [6–12]. Findings in our study were in line with those reported from Western communities. The consistency in findings regarding lifestyle-related behaviors and HRQoL among students from different societies is interesting and meaningful given the heterogeneity of social and cultural contexts, and the different instruments used to assess HRQoL. This suggests the relationship between lifestyle-related behaviors and HRQoL is likely to hold widely for children and adolescents.

Potential mechanisms underlying the associations of lifestyle-related behaviors with HRQoL among children and adolescents have been postulated. PA could improve adolescents’ social functioning and desirability [25], while SB might be associated with social inactivity, loneliness and shyness for adolescents [26, 27]. This might, in part, explain the positive PA-HRQoL and negative ST-HRQoL associations among children and adolescents. With respect to sleep, it has been documented that insufficient TSD or deprivation of sleep could contribute to experiencing fatigue and depression [28–31]. This might partly explain why students with insufficient TSD tended to report lower HRQoL utility scores. In regard to eating behaviors, consumption of energy-dense diets, such as FF and SSB, has been examined to be positively associated with unhappiness, poor sleep, perceived stress and depression [32–34], which might underlie the positive SSB-HRQoL and FF-HRQoL relationships among our study participants.

The joint association of the five investigated lifestyle-related risk behaviors with HRQoL was consistent with the findings from Australian school children [12]. There were additive effects for the five lifestyle-related risk behaviors on HRQoL. This finding has particular public health implications, as it suggests lifestyle-related behavior interventions designed to improve HRQoL for students should target multiple risk behaviors in a campaign in order to optimize the input–output effect.

This is the first study investigating the associations between five classical lifestyle-related behaviors and HRQoL among school-aged children and adolescents in China. There were several strengths of our study. Firstly, participants were randomly selected from all urban, suburban and rural areas, and were representative of local students in Nanjing, a typical Chinese mega-city. Secondly, data were collected from eligible students on scheduled survey dates with the assistance of our research team members and the classroom teachers under the strict quality control, thus a very high response rate (97.6%) was obtained. This indicates the findings will have excellent generalisability. Thirdly, the instrument used to measure HRQoL was validated and the scoring algorithm was developed specially for students in China. Fourthly, five typical lifestyle-related risk behaviors were simultaneously involved in this study. Finally, in addition to associations between individual risk behaviors and HRQoL, the combined association of those five risk behaviors with HRQoL was examined.

Some study limitations also exist. First, information on lifestyle-related behaviors was gathered via self-report, although validated and reliable instruments were used. Second, independent variables distributed abnormally, so they were used as categorical measures. This might imply potential loss of variance in analysis. Third, no causality of the observed associations can be inferred due to the cross-sectional design. Last, some potential confounding factors might not be controlled for in our study, although we have adjusted for participants’ socio-demographic attributes, body weight status and class-level clustering-effects in the analysis. In future, well-designed studies, particularly longitudinal studies and intervention programs, within different cultural and social context are needed to further understand the relationship between lifestyle-related behaviors and HRQoL among children and adolescents worldwide.

In conclusion, with the study based on a representative sample of Chinese school-aged children and adolescents, we provided further evidence that: (1) physical activity and sleep duration were positively associated with HRQoL; (2) sedentary behavior, consumption of sugar-sweetened beverage and fast food were negatively associated with HRQoL; and (3) these five lifestyle-related risk behaviors might exert additive effects on HRQoL. This study has important public health implications that lifestyle and behavior interventions can improve students’ HRQoL, and targeting more risk behaviors in health promotion campaigns will generate more effect on HRQoL among children and adolescents.

## Data Availability

Data is available upon request to corresponding authors.

## Appendix 1

See Table

**Table 4:** Mixed-effects linear regression estimates for mean differences in HRQoL utility scores by gender, school type and residence in this study

	Gender						School type									Residence								
	Boys (N = 2204)			Girls (N = 2184)			Primary (N = 1602)			Junior high (N = 1455)			Senior high (N = 1331)			Rural (N = 2030)			Sub-urban (N = 997)			Urban (N = 1361)		
	Mean	95% CI	*P*	Mean	95% CI	*P*	Mean	95% CI	*P*	Mean	95% CI	*P*	Mean	95% CI	*P*	Mean	95% CI	*P*	Mean	95% CI	*P*	Mean	95% CI	*P*
Physical activity^a^																								
Insufficient	− 0.02	− 0.04, − 0.01	0.008	− 0.03	− 0.05, − 0.01	0.005	− 0.027	− 0.03, 0.00	0.046	− 0.02	− 0.04, − 0.001	0.043	− 0.04	− 0.07, − 0.01	0.010	− 0.02	− 0.04, − 0.01	0.007	− 0.04	− 0.07, − 0.02	0.001	− 0.01	− 0.05, 0.02	0.406
Screen time																								
≥ 2 h/day	− 0.05	− 0.09, − 0.01	0.009	− 0.04	− 0.09, − 0.001	0.047	− 0.08	− 0.13, − 0.03	0.006	− 0.06	− 0.09, − 0.02	0.005	− 0.003	− 0.06, 0.05	0.903	− 0.06	− 0.10, − 0.01	0.009	− 0.01	− 0.07, 0.05	0.765	− 0.04	− 0.11, 0.02	0.163
Sleeping time^b^																								
Insufficient	− 0.06	− 0.08, − 0.04	0.000	− 0.05	− 0.07, − 0.03	0.000	− 0.03	− 0.05, − 0.01	0.025	− 0.08	− 0.11, − 0.05	0.000	− 0.06	− 0.10, − 0.02	0.005	− 0.04	− 0.06, − 0.01	0.004	− 0.08	− 0.12, − 0.04	0.000	− 0.08	− 0.11, − 0.05	0.000
Sugar-sweetened beverage consumption^c^																								
Yes	− 0.01	− 0.03, 0.002	0.085	− 0.04	− 0.06, − 0.02	0.000	− 0.04	− 0.06, − 0.02	0.000	− 0.03	− 0.05, − 0.01	0.001	0.002(0.011)	− 0.02, 0.02	0.883	− 0.02(0.01)	− 0.04, − 0.01	0.003	− 0.03	− 0.06, − 0.01	0.004	− 0.02	− 0.04, 0.001	0.067
Fast food consumption ^c^																								
Yes	− 0.02	− 0.04, − 0.01	0.008	− 0.04	− 0.05, − 0.02	0.000	− 0.03	− 0.05, − 0.01	0.011	− 0.03	− 0.06, − 0.00	0.009	− 0.03	− 0.05, − 0.01	0.020	− 0.04(0.01)	− 0.05, − 0.02	0.000	− 0.03	− 0.05, 0.003	0.073	− 0.03	− 0.06, 0.002	0.061

Mixed-effects linear regression analysis with adjustment for age, gender, school type, residence, parental educational attainment, body weight status and class-level clustering effects

^a^Sufficient physical activity refers to at least 60 min/day moderate-vigorous intensity physical activity plus ≥ 3 days muscle/bone-strengthening; while insufficient physical activity means less than 60 min/day moderate-vigorous intensity physical activity or having no ≥ 3 days muscle/bone-strengthening

^b^Sleeping time: sufficient sleeping time was defined as 10 h/day for children aged 7–13, 9 h/day for children aged 13–16, and 8 h/day for those aged 16–19, based on guidelines for promotion of children and adolescents’ physical activity and fitness by The State Council of China

^c^Sugar-sweetened beverage and fast food consumption was separately categorized into: “no” or “yes” based on the weekly consumption frequency, separately

4.
